# Assessing Habitat Suitability and Connectivity of Black Storks in China: Integrating Species Distribution Models and Landscape Connectivity Analysis

**DOI:** 10.1002/ece3.72177

**Published:** 2025-10-03

**Authors:** Zhiheng Zhang, Jinyu Yang, Xiaohan Yu, Yuerong Jia, Lei Zhang, Dongmei Wan

**Affiliations:** ^1^ Laboratory of Animal Resources and Epidemic Disease Prevention, School of Life Sciences Liaoning University Shenyang China; ^2^ Shanghai University of Finance and Economics Shanghai China; ^3^ School of Basic Medical Sciences Hebei Medical University Shijiazhuang China

**Keywords:** ecological nodes, landscape connectivity, MaxEnt model, species conservation

## Abstract

The black stork ( *Ciconia nigra* ), recognized as a wetland umbrella species and biological indicator, plays a crucial role in maintaining ecosystem balance and biodiversity conservation. However, the black stork faces significant threats from habitat fragmentation and degradation. This study employed the MaxEnt model and landscape connectivity analysis to evaluate suitable habitats for black storks in China, design an ecological corridor network, and identify key ecological nodes. The findings reveal that areas of high habitat suitability are primarily located in North China, the northwestern region of Xinjiang, and the middle and lower reaches of the Yangtze River. The ecological corridor network connects regions between North China and the Yangtze River Basin, forming a rectangular network with vertices in Gansu‐Qinghai, Shanxi‐Beijing‐Tianjin‐Hebei, the lower Yangtze, and Sichuan‐Yunnan Province, respectively, totaling 28,312 km in length. Additionally, four ecological nodes requiring priority protection and management were identified. The study proposes conservation strategies that improve habitat connectivity and ecological functionality to ensure the long‐term stability of black stork populations. Such strategies include prioritizing the protection of highly suitable habitats (e.g., in Shanxi and Hebei), enhancing ecological restoration in the Hexi Corridor, and strengthening the conservation and management of nature reserves by improving ecological connectivity, clarifying functional zoning, and enhancing monitoring and enforcement capacity.

## Graphical abstract

The black stork, a vital wetland species, is threatened by habitat fragmentation, with this study using the MaxEnt model to identify key habitats in North China, Xinjiang, and the Yangtze River Basin. An ecological corridor network of 28,312 km connects these regions, highlighting four critical nodes for prioritized conservation to enhance habitat connectivity and ensure population stability.

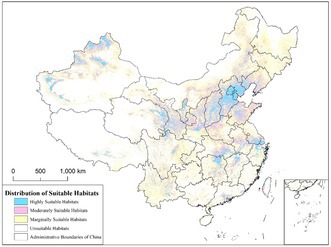

## 1 Introduction

Biodiversity maintains ecosystem functions, enhances resource utilization efficiency, and provides crucial ecosystem services (Duffy 2009). However, with the rapid development of human society, global biodiversity has become increasingly vulnerable, threatened by environmental issues such as habitat fragmentation (Ramírez‐Delgado et al. 2022), resource overexploitation, pollution (Eni et al. 2025), and climate change (de Souza et al. 2025). Among the various environmental issues, habitat fragmentation has emerged as a major threat (Pereira et al. 2010). Continuous habitats are divided into isolated and spatially heterogeneous patches because of human activities (Collinge and Forman 1998; Ewers and Didham 2006). This does not just affect species distribution (Haddad et al. 2015; Keinath et al. 2017) but could significantly increase their risk of extinction as it hinders species dispersal and exacerbates genetic isolation (Wilcox and Murphy 1985; Deb et al. 2019).

**FIGURE 1:**
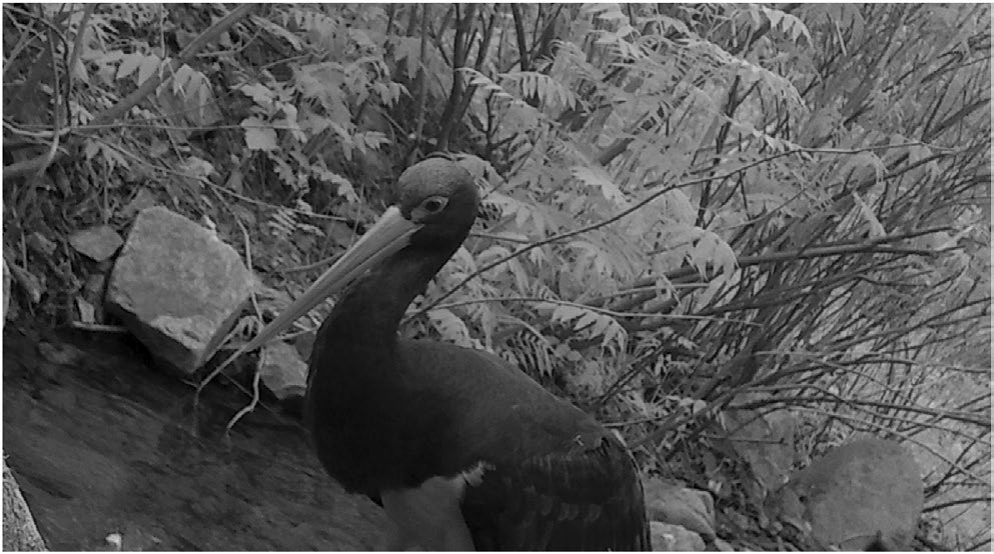
The black stork ( *Ciconia nigra* ).

China is one of the most biodiverse countries in the world (Wang et al. 2020). However, this diversity is at risk from habitat fragmentation caused by aggressive expansion of cities and agriculture, as well as transportation infrastructure networks (Zhou et al. 2021). Wetlands, which are influenced by both aquatic and surrounding terrestrial environments, are hit disproportionately hard (Terrado et al. 2016). In particular, over the past 40 years, China's wetland area has decreased by more than 12% (Mao et al. 2025), severely impacting rare and endangered bird species including the oriental white stork ( *Ciconia boyciana* ) (Kavana et al. 2024), red‐crowned crane ( *Grus japonensis* ) (Li et al. 2025), and crested ibis ( *Nipponia nippon* ) (Sun et al. 2016). The black stork ( *Ciconia nigra* ) (Figure 1), a large and endangered waterbird and an umbrella species in wetland ecosystems (Eaton and Cano‐Alonso 2022), is widely distributed across the Eurasian temperate regions (Gula et al. 2023), with scattered populations in southern Africa (Bobek et al. 2008). The black stork plays a crucial role in maintaining food web balance and assessing the health of wetlands and forests (Eaton and Cano‐Alonso 2022). However, the population of the black stork is at risk due to wetland habitat fragmentation, forcing it to relocate to suboptimal habitats. The shift to these lower‐quality environments increases survival pressure on black storks, as foraging becomes less efficient and energy expenditure during migration rises (Xing et al. 2025). The black stork is currently listed as Least Concern on the IUCN Red List. However, in China, the black stork is designated as a Class I nationally protected species, reflecting a higher conservation priority at the national level. In China, black storks primarily breed in regions north of the Yellow River including northeastern, northern, and northwestern China (Wang et al. 1998), and winter mainly in wetlands along the middle reaches of the Yellow River, the Weihe River and its tributaries, and the Han River basin (Tuohetahong et al. 2023). In the late 20th century, the wild population of black storks in China was estimated to be only around 1000 individuals (Wang et al. 1998), indicating long‐term vulnerability. Moreover, predictive models suggest that suitable habitats for the black stork may continue to shrink in the future under scenarios of land use and climate change (Tuohetahong et al. 2023). These findings underscore the urgent need for targeted habitat conservation and connectivity planning. Research on the black stork in China has primarily focused on behaviors (Xiaojing et al. 2011; Cano Alonso 2013; Freschi et al. 2023) and genetics (Lanzarot et al. 2005; Liu et al. 2016; Liang et al. 2019), with few resources devoted to habitat fragmentation and suitability studies (Tuohetahong et al. 2023). Studies on the mechanisms of habitat fragmentation and connectivity are also scarce, but these are vital aspects in protecting its core habitats and promoting population dispersal and gene flow (Margules and Pressey 2000). Most studies on habitat fragmentation and connectivity are concentrated in Europe and North America, with relatively few conducted in Asia (Fardila et al. 2017). These studies primarily focus on mammals (Clément et al. 2014), and among birds, the emphasis is mostly on passerines (Engler et al. 2017). Large wetland‐associated species like the black stork have received little attention. In China, only one study has addressed the wintering distribution of the black stork (Tuohetahong et al. 2023), and research covering its entire annual cycle remains scarce.

In the global context of habitat fragmentation, the core strategy for species conservation is building ecological networks and enhancing landscape connectivity (Saura et al. 2019; Cao et al. 2020). Consequently, relevant research has largely focused on species distribution prediction (Pearson and Dawson 2003) and landscape connectivity analysis (Rao et al. 2025). Ecological corridors, linear landscapes connecting isolated habitats (Puth and Wilson 2001), are important tools for mitigating habitat fragmentation and strengthening population connectivity (Jia et al. 2023). Building frequently utilized corridors relies on accurate identification of ecological source areas and the establishment of resistance surfaces (Ding et al. 2023). Ecological source areas refer to core patches in a regional ecosystem that have major ecological functions, sustain biodiversity, and provide key habitats (Cao et al. 2022). Resistance surfaces are spatial distribution models reflecting the resistance encountered by species during movement across the landscape, quantifying factors such as land use type, elevation, slope, and aspect (Zhou and Song 2021). Ecological pinch points are the narrowest, most vulnerable, and critical areas for species migration/connectivity within ecological corridors (McRae et al. 2008). Typically, there are few or no alternative pathways there. Once damaged (e.g., through road construction, urbanization, and so forth), this may result in corridor fragmentation, threatening species migration, gene flow, and habitat connectivity. Therefore, they are a priority of habitat protection (Rahimi and Dong 2023). Obstacle points are areas in the landscape that impede species migration, typically caused by human activities (such as roads and cities) or natural factors (such as rivers and mountains) (Wei et al. 2022).

The MaxEnt model has gained widespread recognition among researchers for its high accuracy (Duan et al. 2014), low data volume required, and ease of use (Tsiftsis et al. 2019). It has been successfully applied in habitat suitability modeling for a variety of threatened species, including the Asiatic Black Bear ( *Ursus thibetanus* ) (Su et al. 2021), Snow Leopard ( *Panthera uncia* ) (Aryal et al. 2016), and Crested Ibis ( *Nipponia nippon* ) (Zheng et al. 2018), demonstrating its robustness across different ecological contexts.

In ecological corridor planning, the Linkage Mapper toolkit—an ArcGIS‐based tool built on the least‐cost path model—is widely used in combination with Circuitscape to identify corridors, pinch points, and barrier areas. This integrated approach has been successfully applied in conservation planning for species such as the South China Tiger ( *Panthera tigris amoyensis* ) (Wu and Convertino 2025) and the Yunnan snub‐nosed monkey ( *Rhinopithecus bieti* ) (Li et al. 2017), enabling the design of corridors between core habitats and the detection of critical connectivity bottlenecks (Cao et al. 2020; Zhu et al. 2025). This method supports the selection of corridors with high practical value and helps prioritize areas for restoration or protection (Xu et al. 2022), and is among the most widely adopted tools in ecological corridor construction (McRae et al. 2008; Unnithan Kumar and Cushman 2022).

This study identifies potential core habitats of the black stork ( *Ciconia nigra* ) in China and determines key environmental drivers using the MaxEnt model based on distribution data and environmental variables. Subsequently, the Linkage Mapper tool, in combination with the Circuitscape plugin, is employed to construct an ecological corridor network and identify ecological pinch points and barrier points. The objectives were: (a) to analyze the spatial distribution patterns of potential suitable habitats and identify the dominant environmental factors influencing this distribution and (b) to identify core habitats and ecological corridors, locate pinch points and barrier points within the corridor network, based on which to propose recommendations for habitat restoration and protection. These efforts aim to facilitate inter‐population connectivity and maintain functioning migration routes for the species.

## 2 Materials and Methods

### 2.1 Distribution Data Collection and Processing

In this study, distribution records of the black stork were obtained from three species occurrence databases: eBird (https://ebird.org/home) (Sullivan et al. 2009), the Global Biodiversity Information Facility (GBIF, https://www.gbif.org) (Luo et al. 2021), and iNaturalist (https://www.inaturalist.org) (Van Horn et al. 2018), covering the period from January 2015 to January 2025. To ensure the accuracy of distribution points, only occurrence records with precise geographic coordinates were retained. Specifically, for iNaturalist, only records with accompanying photographs and a spatial uncertainty of less than 5 km were included, and for eBird, only validated (reviewed) records were used. In addition, two field‐verified black stork occurrence points recorded during the same period were also incorporated into the dataset. For habitat suitability analysis, year‐round occurrence data were used to develop MaxEnt models to predict potential suitable habitats of the black stork.

To explicitly account for the seasonal migratory behavior of black storks, we divided the occurrence data into full‐year data and non‐migratory period data. The full‐year data were used to analyze the overall habitat suitability and distribution of black storks across China. Ecological corridors were constructed using only the non‐migratory period data, which includes the breeding season (April–July) (Zhao 2001) and the wintering season (December–February of the following year) (Liu et al. 2013), when black storks are relatively stationary.

**FIGURE 2:**
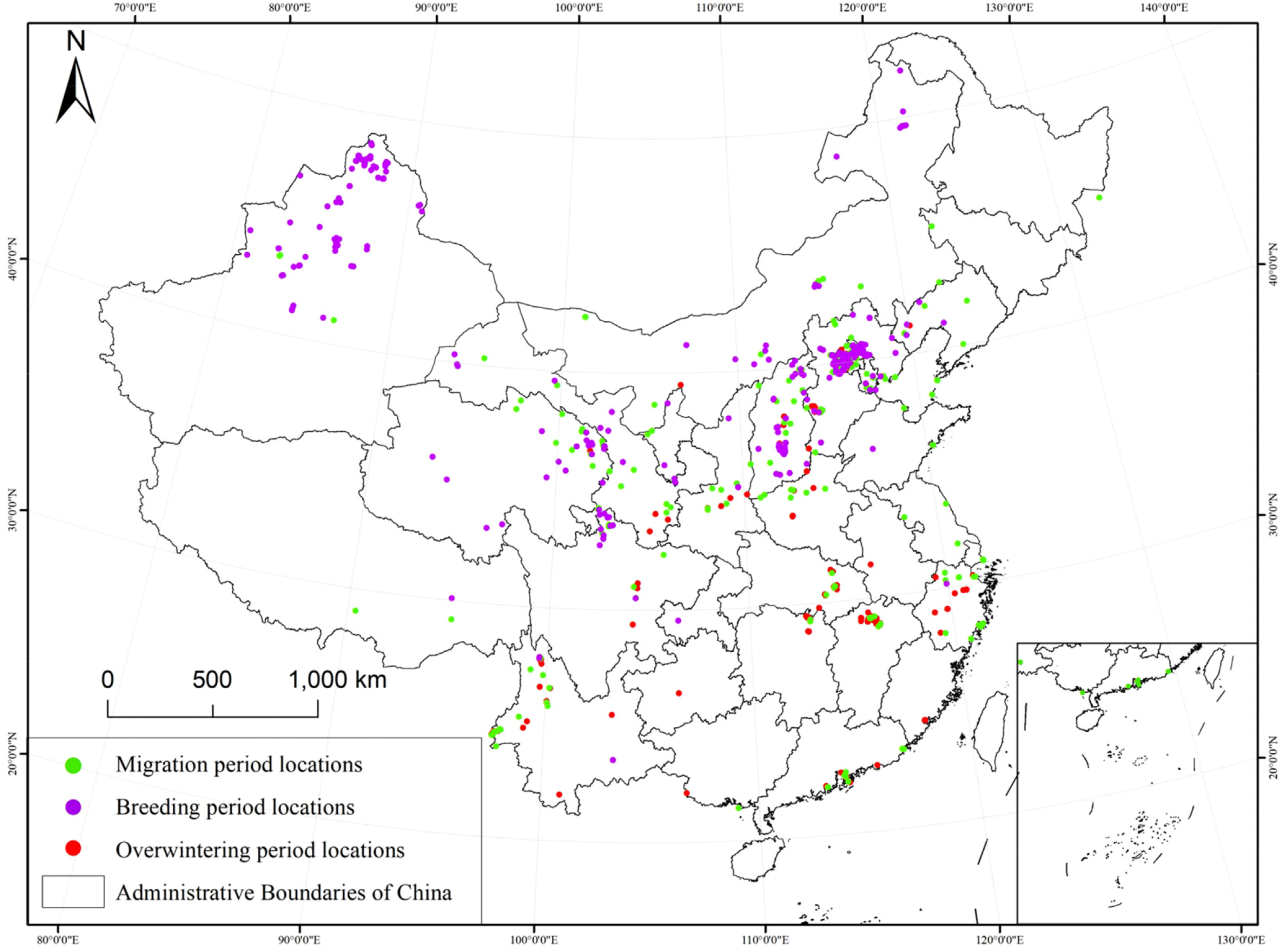
Distribution map of black stork occurrence points. Green dots represent records during the migratory period, purple dots indicate records from the breeding season, and red dots denote records from the wintering season. The sum of occurrence points from the breeding and wintering seasons equals the number of points in the non‐migratory period.

To reduce spatial redundancy among occurrence records, the distribution data were filtered using ENMTools software in combination with climate variables (Warren et al. 2010). Specifically, only one presence point was retained within each ~1 km grid cell (Elith et al. 2006), minimizing spatial autocorrelation and sampling bias. As a result, a total of 719 occurrence records spanning the full year were retained for modeling, with 529 records corresponding to the non‐migratory period (Figure 2).

### 2.2 Acquisition and Processing of Environmental Variables

The altitude data used in this study were from the National Cryosphere Desert Data Center (http://www.ncdc.ac.cn) (Kang 2020). Slope and aspect information were extracted from the altitude data using ArcGIS 10.8.2. Distance‐related variables were sourced from the 1:1,000,000 public version of the basic geographic information data available through the National Catalog Service for Geographic Information (https://www.webmap.cn), which includes water bodies such as lakes, reservoirs, and rivers, roads such as highways and railways, and built‐up areas such as urban blocks, high‐rise building zones, and residential housing. Land cover data were obtained from the National Cryosphere Desert Data Center (http://www.ncdc.ac.cn) (Yang and Huang 2021), and this dataset classified land types into cropland, forest, shrub, grassland, water, snow and ice, barren, impervious, and wetland. NDVI data were sourced from NASA's The Earth Science Data (https://www.earthdata.nasa.gov). Climate variable data (19 variables) were extracted from the WorldClim (https://worldclim.org). Specific environmental variable information is presented in Table 1. The original spatial resolution of all the above environmental variable datasets is 1 km.

**TABLE 1:** Environmental variable names.

Variable type	Name	Interpretation	Whether to use
Topographic variables	Altitude	Altitude (m)	Yes
	Slope	Slope (°)	Yes
	Aspect	Aspect	Yes
Distance‐based variables	Distance to water source	Distance to water source (m)	Yes
	Distance to the roads	Distance to the roads (m)	Yes
	Distance to the urban area	Distance to the roads (m)	Yes
Habitat variables	NDVI	NDVI	Yes
	Land cover	Land cover	Yes
Climate variables	bio1	Annual mean temperature (°C)	No
	bio2	Mean diurnal range [mean of monthly (maxtemp − mintemp)] (°C)	No
	bio3	Isothermality (bio_2/bio_7) (×100)	Yes
	bio4	Temperature seasonality (standard deviation ×100)	No
	bio5	Maximum temperature of warmest month (°C)	No
	bio6	Minimum temperature of coldest month (°C)	Yes
	bio7	Temperature annual range (°C)	Yes
	bio8	Mean temperature of wettest quarter (°C)	No
	bio9	Mean temperature of driest quarter (°C)	No
	bio10	Mean temperature of warmest quarter (°C)	No
	bio11	Mean temperature of coldest quarter (°C)	No
	bio12	Annual precipitation (mm)	No
	bio13	Precipitation of wettest month (mm)	No
	bio14	Precipitation of driest month (mm)	Yes
	bio15	Precipitation seasonality (mm)	Yes
	bio16	Precipitation of wettest quarter (mm)	No
	bio17	Precipitation of driest quarter (mm)	No
	bio18	Precipitation of warmest quarter (mm)	No
	bio19	Precipitation of coldest quarter (mm)	No

**FIGURE 3:**
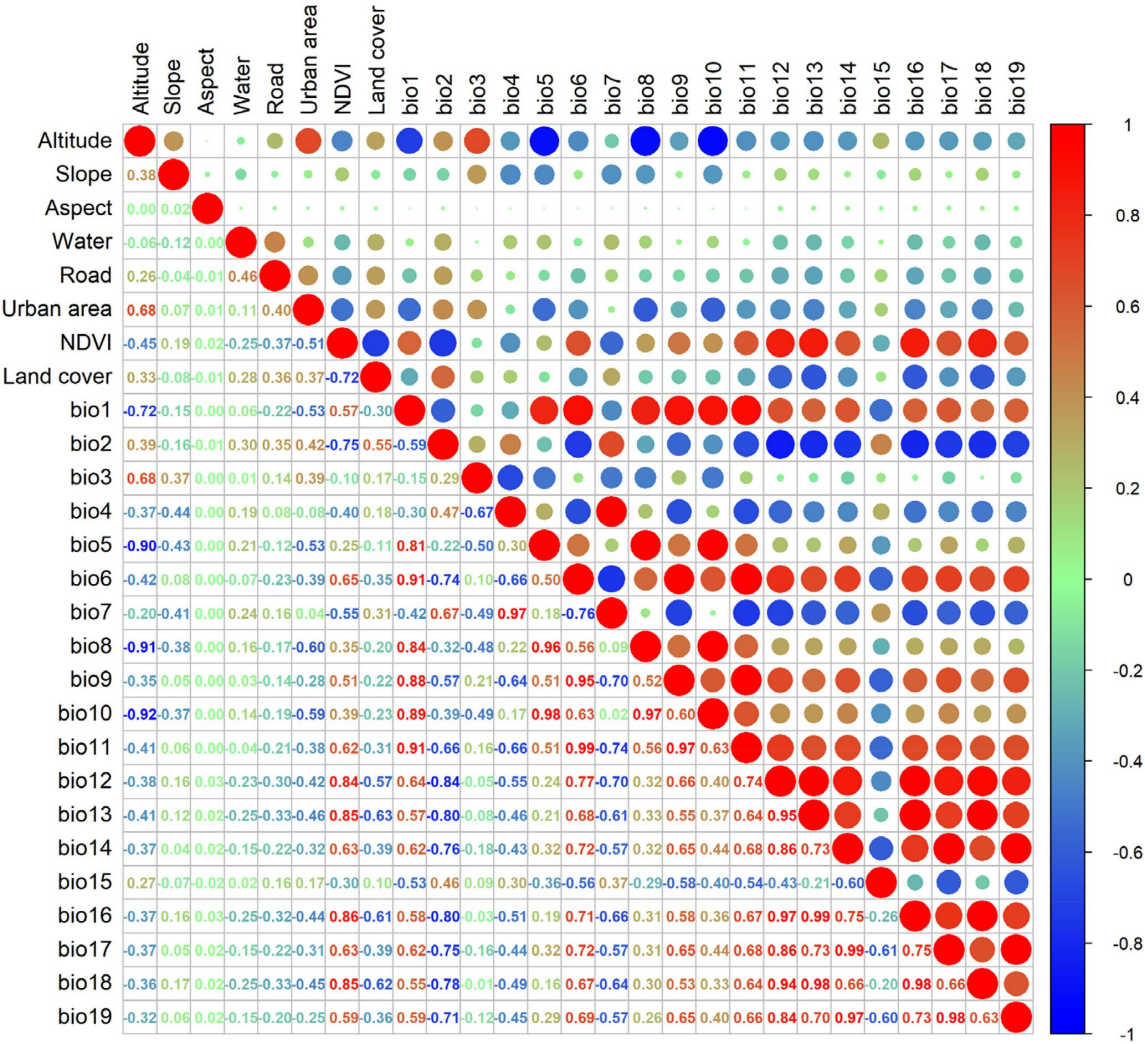
Correlation matrix of environmental variables. In the figure, larger circles indicate stronger correlations between variables. Red represents positive correlations, while blue indicates negative correlations. Among all factors, water denotes the distance to water sources, road represents the distance to roads, and urban area indicates the distance to the urban area.

All environmental variables were standardized using ArcGIS 10.8.2, with the geographic coordinate system set to GCS_WGS_1984 and the projection system set to Krasovsky 1940‐based Albers Equal Area Conic projection. The boundary size and pixel size of all variables were unified. The correlation analysis results between the variables are shown in Figure 3. To reduce the impact of multicollinearity, variables with a correlation ≥ |0.8| and relatively low contribution were removed using R 4.4.0 (Xu et al. 2019). All variables and their usage are listed in Table 1.

### 2.3 MaxEnt Model Parameter Settings

The parameter settings for the MaxEnt model used in habitat suitability prediction and source area reconstruction were as follows: the “create response curves” option was selected to generate response curves illustrating the relationship between environmental variables and species occurrence probability. The jackknife method was employed to evaluate the relative importance of each environmental variable in predicting species distribution (Padalia et al. 2014). The output format was set to Logistic. 75% of the occurrence records were randomly selected to train the model, while the remaining 25% were used to validate model accuracy (Ghazali et al. 2023). The model was replicated 10 times using the bootstrap method (Elith et al. 2011), and all other parameters were kept at their default values.

The predictive performance of the model is evaluated using the Area Under the Curve of the Receiver Operating Characteristic Curve. The AUC value ranges from 0 to 1, with a higher value indicating better model prediction accuracy (Fawcett 2006). When the AUC value is between 0.9 and 1.0, the model's predictive capability is considered excellent (Wan et al. 2019).

### 2.4 Habitat Suitability Zoning and Ecological Corridor Construction

The results from the two MaxEnt model outputs were imported into ArcGIS, and the habitat suitability of the black stork was classified into four categories using the natural breaks method (Bao et al. 2024): unsuitable habitat, low suitability habitat, medium suitability habitat, and high suitability habitat.

#### 2.4.1 Distribution of Suitable Habitats and Its Influencing Factors

In this study, R 4.4.0 was used to enhance the Receiver Operating Characteristic curve, jackknife plot, and variable response curve. ArcGIS 10.8.2 was employed for the visualization of the black stork's suitable habitat distribution.

#### 2.4.2 Ecological Corridors, Pinch Points, and Barrier Points

Although the maximum migration distance of black storks in Europe and Africa can exceed 2000 km (Chevallier et al. 2013), the longest recorded migration distance of black storks in China is currently 956 km (Wang et al. 2018). To avoid functional failure of ecological corridors caused by exceeding the species' migration capacity, the maximum corridor length threshold in this study was set to 1500 km. In addition, to identify ecological pockets and barrier points, the natural breaks classification method was applied to divide these features into four levels, with the highest levels of current density and resistance values selected as the final ecological pockets and barrier points (Peng et al. 2018). Considering that the black stork has an activity range of approximately 20 km (Yao et al. 2009), search radii for barrier point identification were set at 10, 15, and 20 km to ensure both spatial analytical accuracy and ecological relevance.

The inverse of the Habitat Suitability Index (HSI) during the breeding and wintering periods was used as the resistance surface, with resistance values calculated using the formula 1/HSI (LaRue and Nielsen 2008). Areas classified as highly suitable habitats were designated as ecological sources for corridor construction (Tian et al. 2023). However, as there is no definitive record of the minimum habitat area required for black storks, this study adopted the core habitat area of a closely related species, the white stork ( *Ciconia ciconia* ), approximately 50 km^2^ (Zurell et al. 2018), as the minimum threshold for ecological source areas.

The Linkage Pathways Tool within the Linkage Mapper toolbox was used to construct ecological corridors for the black stork in China (Dickson et al. 2013). In addition, ecological pinch points and barrier points along the corridors were identified using the Pinchpoint Mapper and Barrier Mapper tools in Circuitscape (Zhou et al. 2023). Spatial visualizations of ecological corridors, pinch points, and barrier points were all completed in ArcGIS 10.8.2.

## 3 Results and Analyses

### 3.1 Accuracy of the MaxEnt Model

**FIGURE 4:**
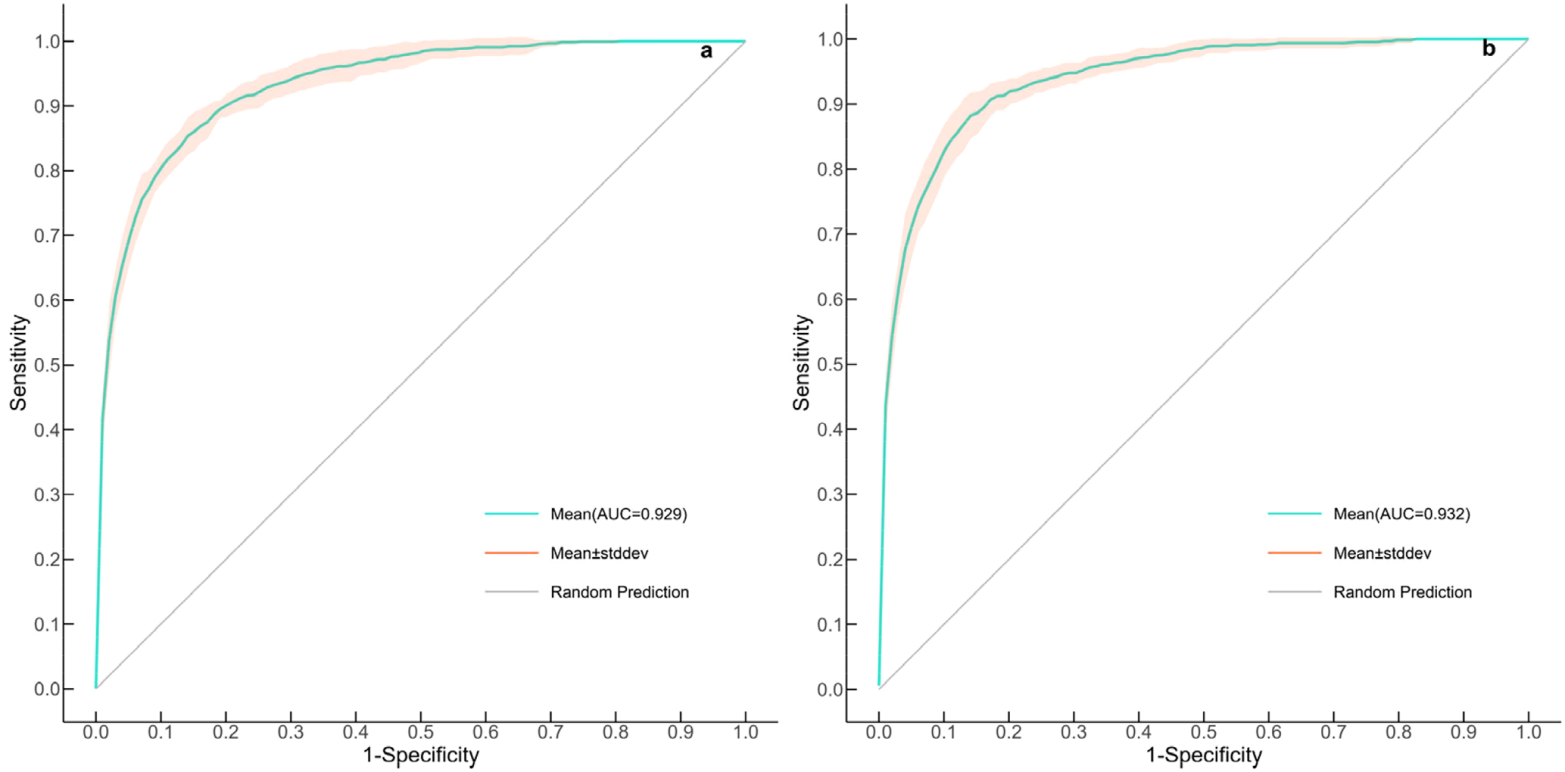
ROC curves. (a) Model based on occurrence records from all seasons; (b) model based on occurrence records from the non‐migratory period.

The ROC curve of the habitat suitability prediction model based on black stork occurrence records from all four seasons is shown in Figure 3 (Figure 4a), with an average AUC value of 0.929. The ROC curve of the model based on non‐migratory period records is shown in Figure 4 (Figure 4b), with an average AUC value of 0.932. Both models yielded AUC values greater than 0.9, indicating excellent predictive performance and demonstrating that the model is suitable for identifying suitable habitats and ecological source areas.

### 3.2 Contribution Rates of Environmental Variables

**FIGURE 5:**
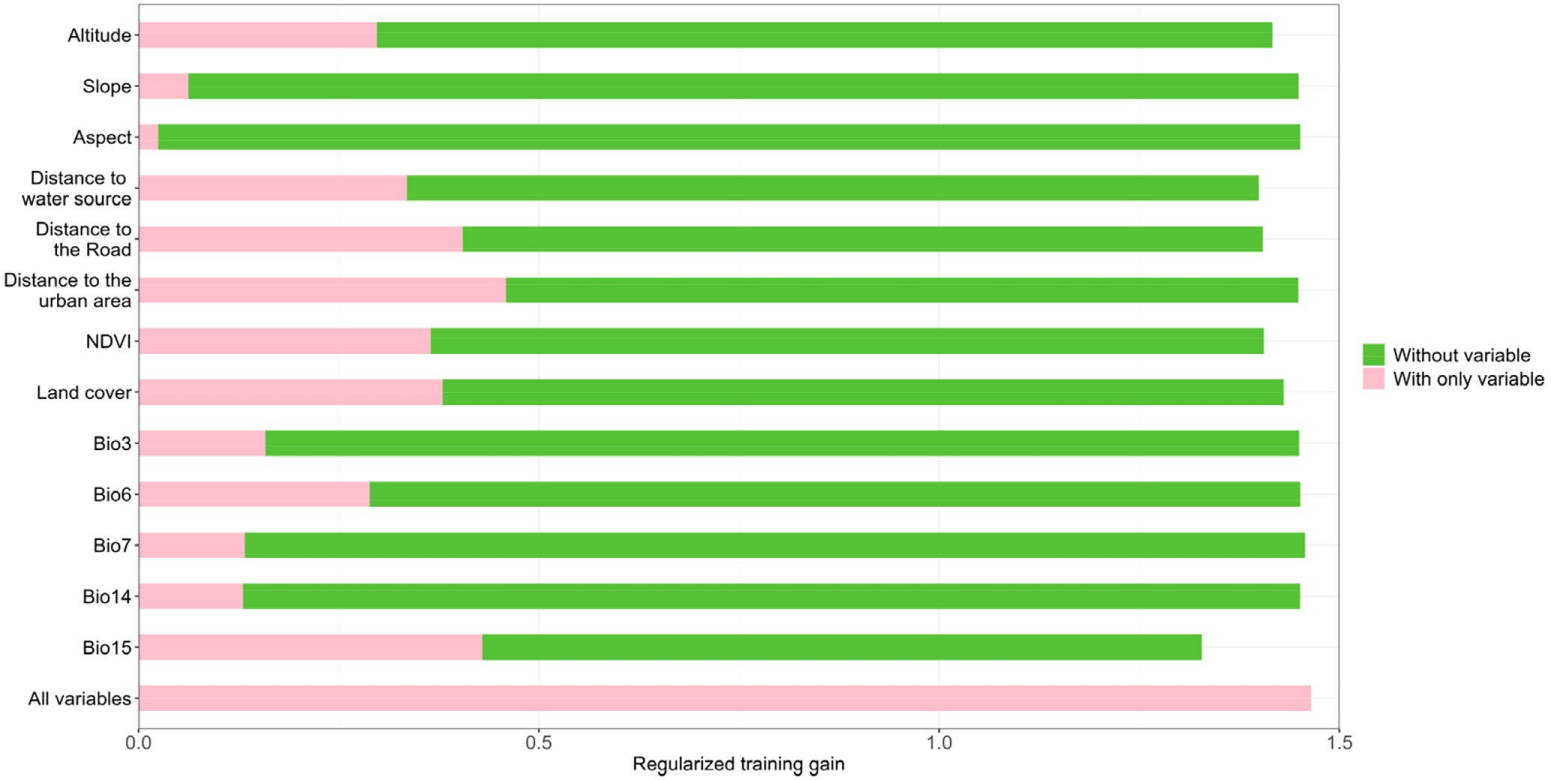
Jackknife test results based on year‐round data.

The Jackknife test was used to identify the key environmental variables with the strongest explanatory power for the distribution of suitable habitats, as shown in Figure 5. The regularized training gain for the distance to built‐up areas was significantly higher than that of other environmental variables, indicating that this factor had the greatest influence on the distribution of black storks. The remaining variables, in descending order of importance, were bio15, distance to roads, land cover type, NDVI, distance to water bodies, altitude, bio6, bio3, bio7, bio14, slope, and aspect. In contrast, the terrain variables—slope and aspect—as well as precipitation of the driest month (bio14) had relatively low regularized training gain values, suggesting a lesser influence on black stork distribution.

**FIGURE 6:**
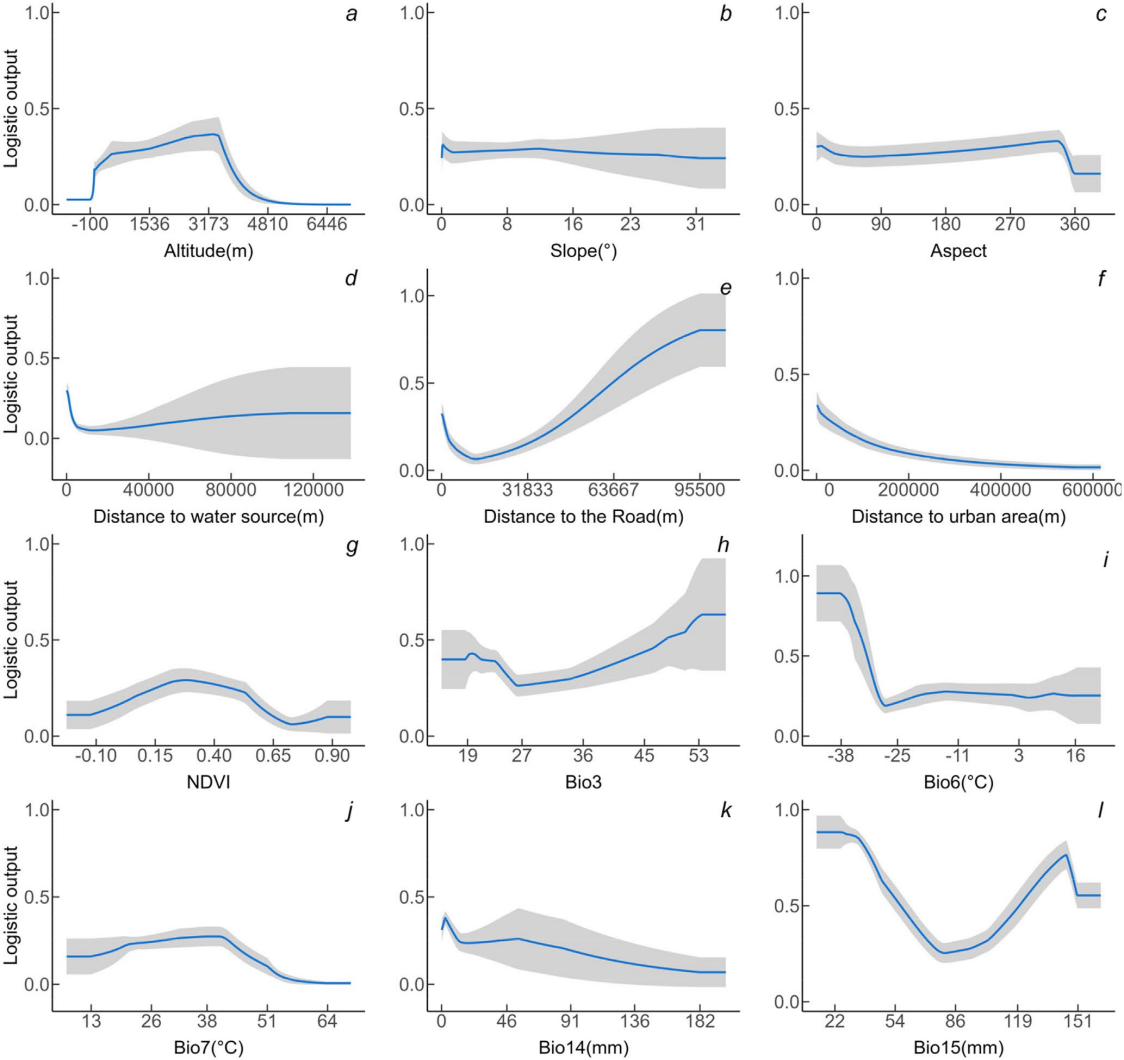
Response curves for continuous environmental variables. The blue lines represent the mean response from 10 replicate MaxEnt runs, and the gray shaded areas indicate the mean ± one standard deviation.

**FIGURE 7:**
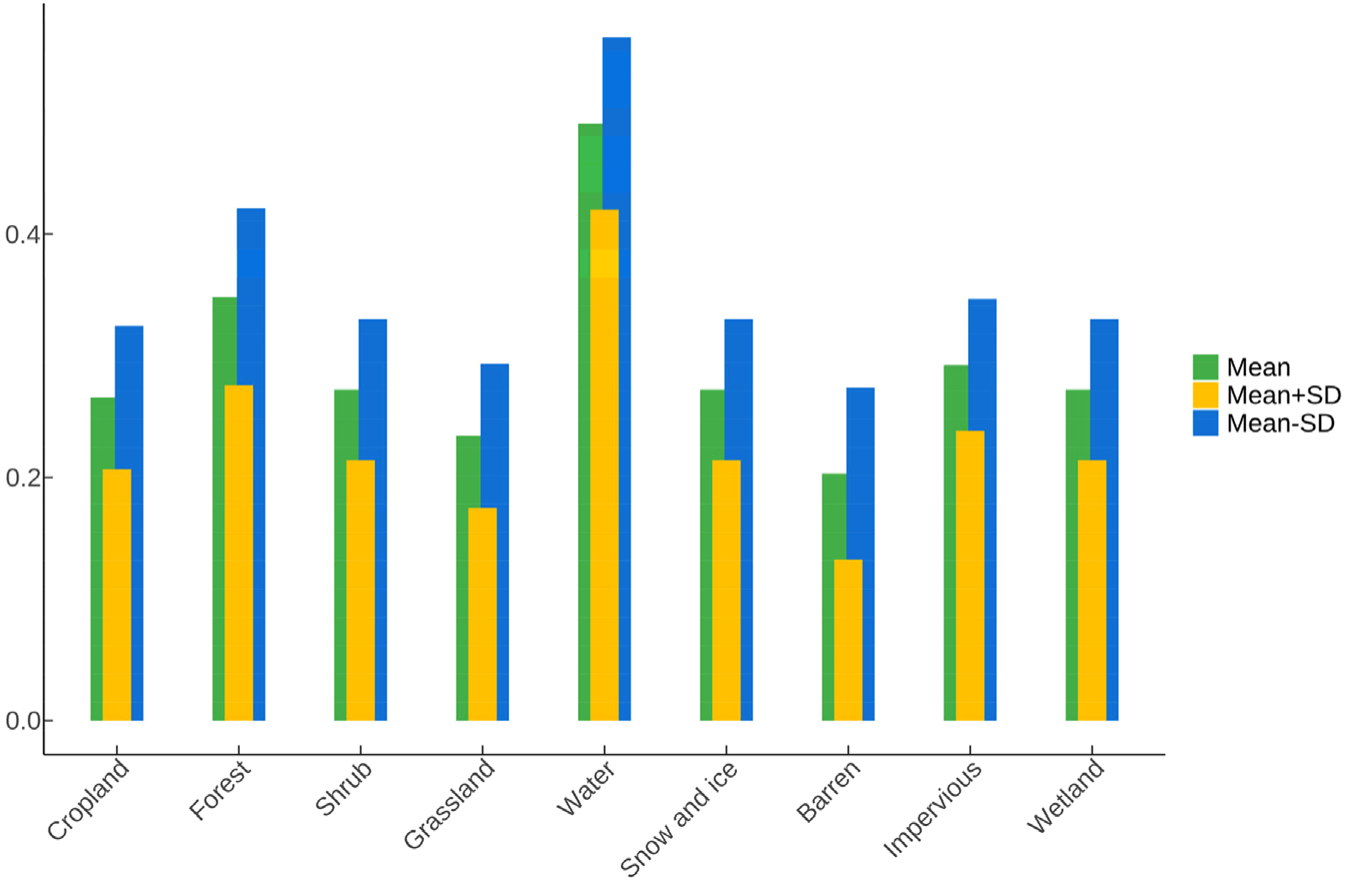
Response curves for categorical environmental variables. Green bars show the mean response across 10 replicate MaxEnt runs, yellow bars indicate the mean plus one standard deviation, and blue bars indicate the mean minus one standard deviation.

The black stork shows a significant preference for mid‐to‐low elevations, with a tendency to select areas between 1000 and 3500 m above sea level (Figure 6a). There is no clear preference for slope or aspect (Figure 6b,c). Among the distance‐related factors, the black stork prefers habitats that are close to water sources and built‐up areas while avoiding roads (Figure 6d–f). The black stork's distribution probability is negatively correlated with the distance to water sources and built‐up areas, and positively correlated with the distance to roads. Regarding vegetation cover, the black stork prefers areas with moderate vegetation index values (Figure 6g). In terms of climate factors, both temperature and precipitation influence the distribution probability of the black stork (Figure 6h,i–l). Among land use types, water bodies have the most significant impact on the distribution of the black stork, while the influence of other land use types is relatively consistent (Figure 7).

### 3.3 Distribution of Suitable Habitats for the Black Stork

**FIGURE 8:**
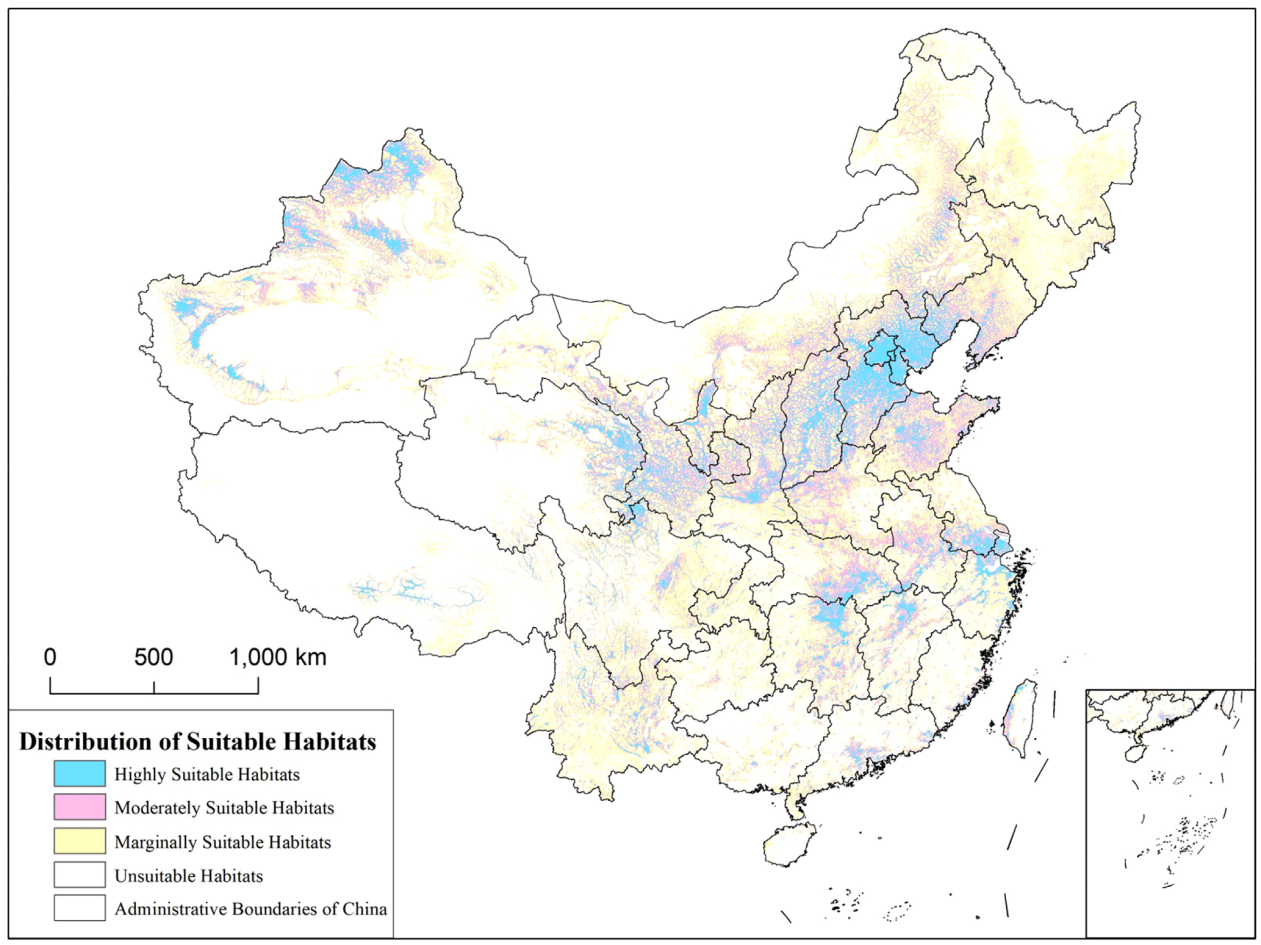
Distribution of suitable habitats for the black stork.

The distribution of suitable habitats for the black stork in China is shown in Figure 8. The area of highly suitable habitat reaches 519,269 km^2^, accounting for approximately 5% of the country's total land area. These highly suitable areas are mainly concentrated in North China, the northwestern part of Xinjiang, and the middle and lower reaches of the Yangtze River. Moderately suitable areas are primarily located around the highly suitable zones, while marginally suitable areas are mainly distributed on the periphery of the moderately suitable zones and in Northeast China.

### 3.4 Ecological Corridors

**FIGURE 9:**
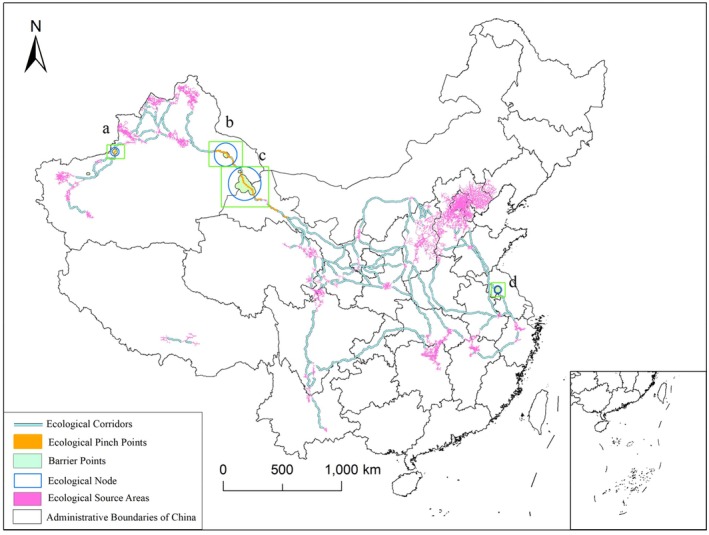
Ecological corridors, ecological pinch points, barrier points, and ecological nodes. The labels a, b, c, and d represent different ecological node regions.

A total of 60 ecological source areas were identified, along with 109 ecological corridors, with a combined length of 28,312 km. The longest corridor measures 1410.80 km, while the shortest spans just 1.32 km (Figure 9). The spatial distribution of ecological corridors exhibits distinct regional patterns, primarily concentrated between northern China and the Yangtze River Basin. These corridors form a rectangular ecological network with four key nodes: the Gansu–Qinghai region, the Shanxi–Beijing‐Tianjin‐Hebei region, the lower reaches of the Yangtze River, and the Sichuan–Yunnan region. This network provides critical ecological pathways for the migration of the black stork, particularly the corridors between the Gansu–Qinghai and Shanxi–Beijing‐Tianjin‐Hebei regions, which represent the most densely connected ecological zones. In contrast, ecological corridors in the northwestern region are relatively sparse. Xinjiang is connected to Gansu via a single corridor located in the Hexi Corridor (hereafter referred to as the Hexi Corridor), which serves as a key route linking the black stork's ecological source areas in northwest China with those in central and east China. Additionally, only one ecological corridor was identified in the Tibet region, connecting two ecological source areas.

### 3.5 Ecological Pinch Points and Barriers

Based on the natural breaks classification method, a total of 75 ecological pinch points were identified. Among them, 60 were concentrated along the Hexi Corridor, while the remaining 15 were located between ecological source areas within Xinjiang. In addition, six ecological barrier points were detected including three along the Hexi Corridor, two within ecological corridors in Xinjiang, and one in the ecological corridor of Jiangsu Province (Figure 9). To facilitate the development of conservation and management strategies for the black stork, both ecological pinch points and barrier points were collectively referred to as ecological nodes. By clustering and integrating adjacent pinch points and barriers, four ecological nodes were ultimately identified. These nodes are located along ecological corridors in Xinjiang, the Hexi Corridor, and Jiangsu Province (Figure 9).

## 4 Discussion

### 4.1 Distribution of Suitable Habitats for the Black Stork and Its Influencing Factors

The habitat selection of the black stork is influenced by multiple environmental factors, and the black stork's distribution in China shows significant regional characteristics (Figures 6, 7, 8). High‐suitability habitats are mainly concentrated in the Yangtze River Basin, North China, and the northwestern region of Xinjiang. Based on environmental variable analysis, the black stork prefers areas of moderate to low elevation, which generally have a mild climate (Pu et al. 2025) and moderate vegetation cover (Zhang, Li, et al. 2022), providing sufficient concealment and stable food resources. In China, the black stork typically nests in mountain or cliff areas during the breeding season (Lee et al. 2023), and the abundant mountain ranges and complex terrain in North China and northwest Xinjiang meet the stork's need for concealed nesting sites. Additionally, studies show that the core activity areas of the black stork are often located within 2500 m of water sources (Tuohetahong et al. 2023). Thus, ecosystems with rich water resources could be good habitat options for black storks such as the lake wetland systems of the Yangtze River Basin, the Yellow River system, and the glacial meltwater networks in northwestern Xinjiang. These water bodies provide rich foraging sites for the black storks. Furthermore, the black stork's response to human activity is dualistic. On one hand, the black stork's distribution probability is negatively correlated with distance from roads, reflecting its tendency to avoid areas with high human disturbance, which is consistent with the findings of Chevallier et al. (2010), who also found that the black stork tends to avoid dense human‐activity areas. On the other hand, despite strong human disturbances and alterations in the natural environment, the black stork is capable of utilizing agricultural landscapes and artificial wetlands as supplementary foraging sites. This demonstrates a habitat selection trade‐off mechanism, reflecting its adaptability to human‐modified landscapes while still depending on natural habitats (de Resende et al. 2024). In summary, the distribution of the black stork is jointly determined by proximity to water sources, elevation, terrain conditions, climate suitability, and human disturbance. The environmental characteristics of high‐suitability habitats are highly aligned with those in the stork's breeding and wintering habits.

### 4.2 Ecological Corridors, Pinch Points, and Barrier Points for the Black Stork

The spatial distribution of ecological corridors and their driving mechanisms is influenced by the combined effects of natural geography, ecological source area patterns, and human activities. For example, a dense rectangular network of ecological corridors was formed between North China and the Yangtze River Basin because there are natural geographic advantages here (the North China Plain has flat terrain and the Yangtze River has intricate tributary systems), which provides a physical foundation for species migration. Moreover, the dense distribution of ecological source areas within these basins reduces the distance between source areas, thereby decreasing the resistance to corridor construction. In other cases, corridors benefited from regional ecological policies such as the establishment of the Beijing‐Tianjin‐Hebei ecological barrier (Liu et al. 2008) and the ecological protection work along the Yangtze River Economic Belt (Peng and Yu 2024). These endeavors have effectively mitigated the impact of urbanization on corridor fragmentation. It showcased that human activities, when properly guided and planned, can make positive ecological impacts. In contrast, the single remaining corridor from Xinjiang to central and east China highlights the ecological fragility of the northwest region. Although the Hexi Corridor serves as a transitional zone and provides a migration route, the area is characterized by sparse rainfall and intense evaporation (Zhu et al. 2022), resulting in severe fragmentation of ecological source areas. Furthermore, factors such as poor soil quality (Luo et al. 2024; Ting et al. 2025), air pollution (Guan et al. 2019), and excessive water resource exploitation (Wang et al. 2025) further weaken the connectivity of this potential corridor, making it a critical bottleneck on the migration route connecting the black stork's eastern and western populations.

To restore the connectivity of black stork ecological nodes, we analyzed key factors influencing their distribution (Figures 6 and 7) and found that the formation of ecological nodes was mainly affected by the barrier effect of mountains and land use types. These factors spatially blocked migration paths and restricted the habitat distribution of the black stork, creating obstacles along the corridors.

**FIGURE 10:**
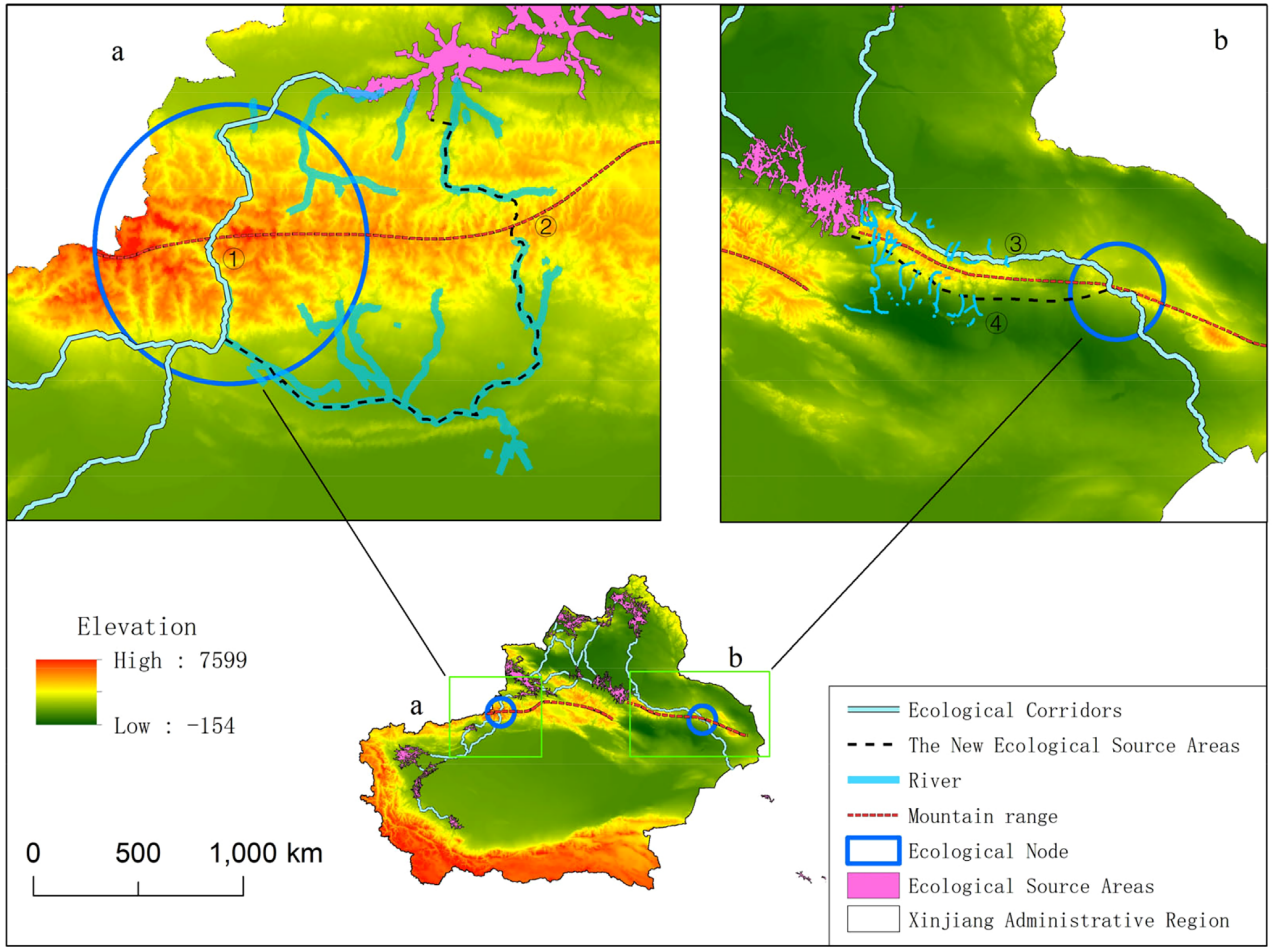
Ecological nodes a and b. ①③ represent the original locations of the two regions' ecological corridors, while ②④ represent the locations of the redirected ecological corridors.

Ecological nodes c and d are primarily affected by the barrier effect of the Tianshan Mountains and the Bogda Mountains (Figure 10a). The main peak of the Tianshan Mountains has an elevation between 4000 and 6000 m (Aizen et al. 1997), and the average elevation of the Bogda Mountains is as high as 5445 m (Du et al. 2021). The mountainous barriers formed by these two mountain ranges increase the migration cost of birds (Henningsson and Alerstam 2005). The physical barriers created by the complex mountainous terrain pose significant obstacles to bird migration, forcing them to expend more energy to detour or ascend. Additionally, the mountains can alter local wind conditions, reducing wind support during bird flight and increasing the risk of energy expenditure during migration (Aurbach et al. 2018). Given that birds instinctively adjust their migration strategies when encountering topographic obstacles (Biebach et al. 2000), this study proposes an ecological corridor optimization plan to address the issues black storks face in this migration route. As shown in Figure 10, the two new ecological corridor routes (indicated by black dashed lines) provide better water resource supply compared with the original ecological nodes c and d, while avoiding the high mountain barriers, thus effectively meeting black storks' needs along the migration. Therefore, by protecting water resources along the new corridor routes, ecological conservation efforts can be further promoted, enhancing the sustainability of migration.

**FIGURE 11:**
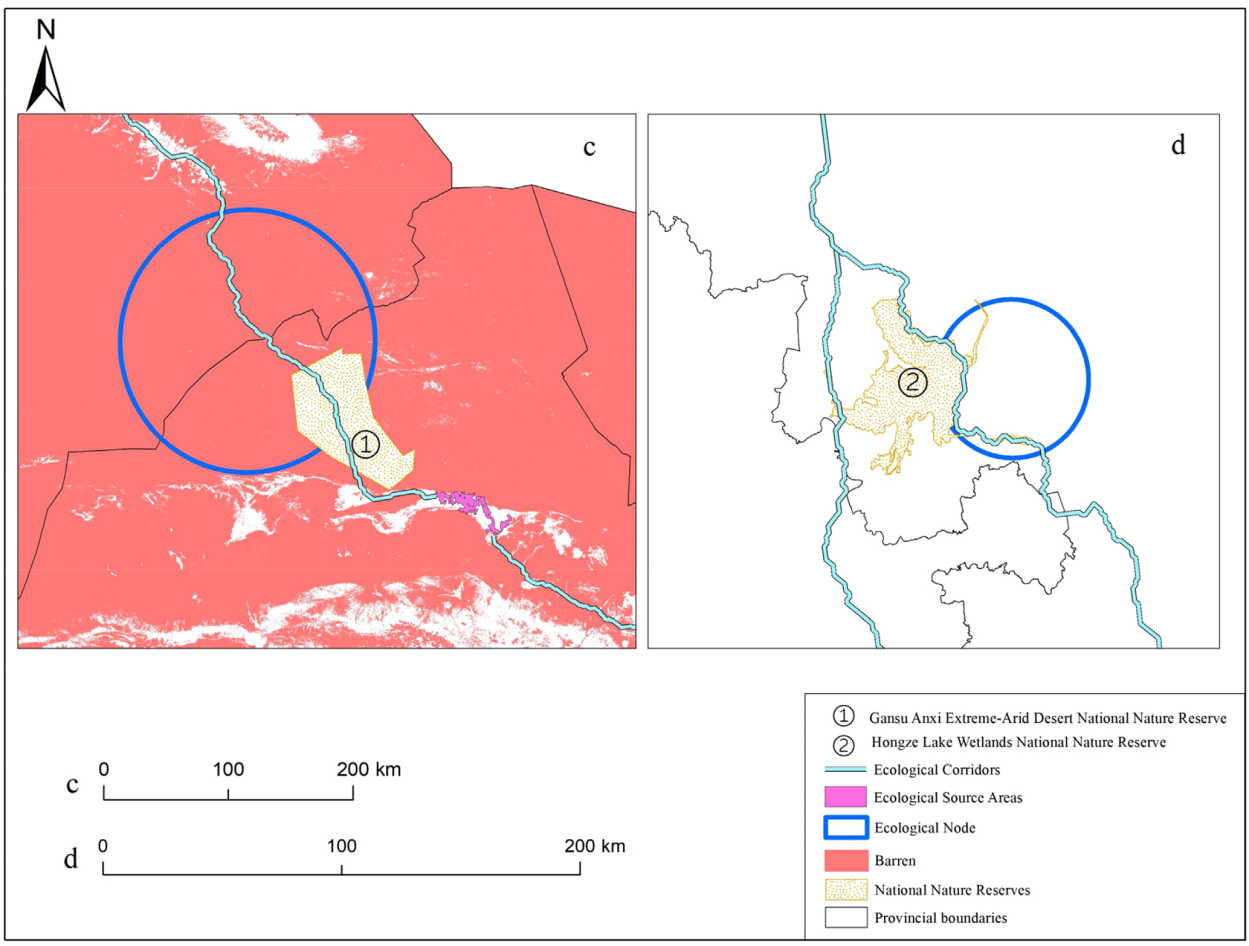
Ecological nodes c and d. ① represents the Gansu Anxi Extreme Arid Desert National Nature Reserve, and ② represents the Su Sihong Hongze Lake Wetland National Nature Reserve.

The main issue affecting ecological node c is the extensive barren land (Figure 11a) and low vegetation coverage (He et al. 2024), which is highly detrimental to the migration and stopover of black storks. Additionally, the Hexi Corridor is located in an arid climate zone with annual precipitation of only 110–350 mm (Duan et al. 2022; Aizen et al. 1997), further exacerbating the difficulty of migration. Therefore, constructed wetlands are recommended, for example in the Gansu Anxi Extreme Arid Desert Conservation Area, to conserve water resources and support wildlife habitat such as providing stopover points for migratory species including the black stork. These treatment systems have the additional benefit of protecting China's afforestation and desertification‐combating effort in its northwest (Qi et al. 2023). In contrast, the restoration of ecological node d is relatively simple, as it is not only close to the Su Sihong Hongze Lake Wetland National Nature Reserve (Figure 11b) but also has alternative corridors nearby. Therefore, this node does not hinder the migration of the black stork. For the isolated population in Tibet, we recommend protecting this small population separately to prevent its extinction.

### 4.3 Protection Recommendations for the Black Stork

The black stork is a wading bird that highly depends on wetlands rich in aquatic organisms for foraging (Clancy 2011), and it also requires quiet, continuous, and safe stopover habitats to support its long‐distance migration (Anderwald et al. 2024). Its ecological habits determine that protecting high‐quality wetlands, restoring key migration corridors, and strengthening water body management are crucial to ensuring its survival and migratory success. Based on the habitat suitability models and ecological corridor connectivity analyses conducted in this study, three targeted conservation recommendations are proposed. First, protect the high‐suitability areas for the black stork. These protection measures include building ecological buffer zones around the Beijing‐Tianjin‐Hebei urban cluster, protecting corridors from being squeezed by urbanization, and strengthening the dynamic monitoring and protection of wetlands in the Yellow River and Yangtze River basins. In addition, to ensure critical source areas are connected, ecological red lines should be established in key ecological nodes such as the northern oasis belt of the Tarim Basin in Xinjiang. Ecological red lines are inviolable boundaries designated by China to safeguard ecological security by strictly limiting development activities in areas with important ecological functions, high biodiversity, or ecological fragility (Zhang, Lin, et al. 2022), in order to maintain ecosystem integrity. The goal is to balance biodiversity conservation with agricultural expansion and energy development. Second, prioritize the protection of the Hexi Corridor in the northwest region, with a focus on enhancing ecological restoration. Wetland restoration and vegetation reconstruction should be carried out to provide better foraging and stopover habitats for the black stork. Third, strengthen the management and protection of rivers and other water bodies in existing nature reserves near ecological corridors to provide safe stopover points and foraging areas for black storks. In conclusion, the integrated conservation strategies proposed—encompassing targeted habitat protection, the establishment of ecological corridors, and strengthened management of key ecological nodes—are essential for sustaining the connectivity and ecological integrity of black stork populations across China. These multifaceted measures not only mitigate the adverse impacts of habitat fragmentation and human disturbances but also provide a robust framework to support the species' long‐term viability and resilience in the face of ongoing environmental change.

Several limitations should be acknowledged in this study. First, all species occurrence data were derived from online databases without support from field surveys, which may reduce the reliability of the dataset and introduce potential biases. Future studies could utilize GPS telemetry data for monitoring, which would not only help reduce data biases but also more accurately capture the complex movement behaviors of species such as the black stork. For example, black storks are capable of flying 30–40 km from their core habitats to forage, indicating that habitat connectivity depends not only on spatial proximity. Therefore, incorporating telemetry or tracking data would more effectively reflect species' movement patterns and thus optimize ecological corridor design. Second, key parameters such as the migration distance of black storks and the size of ecological source areas were inferred from literature rather than measured directly, which may affect the precision of habitat suitability and connectivity analyses. Third, environmental variables such as land use, NDVI, and climate are subject to seasonal variation. However, in constructing ecological corridors, we relied on occurrence records from the non‐migratory season and annual average environmental data. This temporal mismatch may overlook important seasonal dynamics and represent a notable limitation in accurately simulating habitat connectivity across different life‐history stages. Future research should integrate field‐based data collection and time‐specific environmental layers to improve the robustness and ecological validity of the models.

## 5 Conclusion

The suitable habitats of the black stork in China are primarily distributed in the Yangtze River Basin, North China, and the northwest region of Xinjiang, where abundant water resources and favorable terrain support its breeding and migration needs. This study found that black storks are most vulnerable to the impacts of urbanized areas, highlighting the need for effective management and regulation of human activities. Through habitat suitability modeling and ecological corridor analysis, we identified key ecological source areas and migration corridors for the species, with a particular emphasis on the priority protection of the Hexi Corridor in northwest China to enhance population connectivity and ecological stability. This research provides scientific guidance and practical strategies for the conservation of the black stork and the development of ecological corridors.

## Author Contributions

**Zhiheng Zhang:** conceptualization (equal), data curation (equal), formal analysis (equal), methodology (equal), visualization (equal), writing – original draft (equal), writing – review and editing (equal). **Jinyu Yang:** data curation (equal), visualization (equal). **Xiaohan Yu:** data curation (equal). **Yuerong Jia:** data curation (equal). **Lei Zhang:** conceptualization (equal), supervision (equal), writing – review and editing (equal). **Dongmei Wan:** conceptualization (equal), supervision (equal), writing – review and editing (equal).

## Conflicts of Interest

The authors declare no conflicts of interest.

## Supporting information


**Data S1:** ece372177‐sup‐0001‐Supinfo1.rar.

## Data Availability

Our bird site data come from eBird (https://ebird.org/home), GBIF (GBIF, https://www.gbif.org), and iNaturalist (https://www.inaturalist.org). Environmental variable data come from the National Cryosphere Desert Data Center (http://www.ncdc.ac.cn), the National Catalog Service for Geographic Information (https://www.webmap.cn), NASA's Earth Science Data (https://www.earthdata.nasa.gov), and WorldClim (https://worldclim.org).
